# *In Vitro* Characterization of Human Adenovirus Type 55 in Comparison with Its Parental Adenoviruses, Types 11 and 14

**DOI:** 10.1371/journal.pone.0100665

**Published:** 2014-06-23

**Authors:** Juan Liu, Qing-Gong Nian, Yu Zhang, Li-Juan Xu, Yi Hu, Jing Li, Yong-Qiang Deng, Shun-Ya Zhu, Xiao-Yan Wu, E-De Qin, Tao Jiang, Cheng-Feng Qin

**Affiliations:** 1 Department of Virology, State Key Laboratory of Pathogen and Biosecurity, Beijing Institute of Microbiology and Epidemiology, Beijing, China; 2 Department of Transfusion Medicine, General Hospital of PLA Air Force, Beijing, China; University of Georgia, United States of America

## Abstract

Human adenovirus type 55 (HAdV-B55) represents a re-emerging human pathogen, and this adenovirus has been reported to cause outbreaks of acute respiratory diseases among military trainees and in school populations around the world. HAdV-B55 has been revealed to have evolved from homologous recombination between human adenovirus type 14 (HAdV-B14) and type 11 (HAdV-B11), but it presents different clinical manifestations from parental virus HAdV-B11. In the present paper, we report the distinct biological features of HAdV-B55 in comparison with the parental viruses HAdV-B11 and HAdV-B14 in cell cultures. The results showed that HAdV-B55 replicated well in various cells, similar to HAdV-B11 and HAdV-B14, but that its processing had a slower and milder cytopathic effect in the early stages of infection. Viral fitness analysis showed that HAdV-B55 exhibited higher levels of replication in respiratory cells than did either of its parents. Cytotoxicity and apoptosis analyses in A549 cells indicated that HAdV-B55 was less cytotoxic than HAdV-B11 and HAdV-B14 were and induced milder apoptosis. Finally, thermal sensitivity analysis revealed that HAdV-B55 exhibited lower thermostability than did either HAdV-B11 or HAdV-B14, which may limit the transmission of HAdV-B55 in humans. Together, the findings described here expand current knowledge about this re-emerging recombinant HAdV, shedding light on the pathogenesis of HAdV-B55.

## Introduction

Human adenovirus (HAdV) infections have been recognized for decades as an important cause of acute respiratory disease (ARD) [1]. Human adenovirus type 55 (HAdV-B55) belongs to the HAdV-B family, and this adenovirus was recently recognized as a re-emerging respiratory pathogen [2], [3]. HAdV-B55 has been associated with both mild and severe clinical disease, presenting with high fever and respiratory symptoms such as cough, myalgia and sore throat [2], [4]. The virus has been reported to have caused outbreaks of ARD among military trainees in Turkey, Spain and Singapore since 1969 and in school populations in China [4]–[8]. These outbreaks usually spread quickly but are only associated with occasional mortality.

Bioinformatics analysis demonstrated that HAdV-B55 evolved from an intertypic recombination event in the hexon gene between HAdV-B11 and HAdV-B14, producing a new serotype [2], [3]. Both HAdV-B14 and HAdV-B11 belong to the subspecies HAdV-B2. HAdV-B14 has been described in association with outbreaks of ARD, with high rates of illness and death, in military and civilian populations in the United States, China and Europe [9]–[12]. Severe respiratory infections caused by HAdV-B14p1, an emerging variant of HAdV-B14, have been recorded in the United States and Europe since 2006 [10], [11], [13], [14]. Interestingly, although HAdV-B14 and HAdV-B55 commonly cause respiratory tract infections, HAdV-B11 instead generally leads to kidney and urinary tract infections. Genome recombination plays an important role in the evolution of HAdVs [15]. Although the major capsid protein hexon is a hot spot for homologous recombination and critical for infection by HAdVs [16], [17], the potential effect of homologous recombination in the hexon gene on the tropism changes and disease severity of HAdV-B55 is currently not well understood.

In the current study, the in vitro biological characteristics of HAdV-B55 were investigated and compared with those of HAdV-B11 and HAdV-B14. Our results describe the distinct biological features of HAdV-B55 in comparison with its parental viruses, HAdV-B11 and HADV-B14. These findings expand current knowledge about this re-emerging recombinant HAdV, shedding light on the potential pathogenesis of HAdV-B55.

## Materials and Methods

### Cells

The human lung cell line A549 (American Type Culture Collection (ATCC) catalog # CCL-185), the human cervical cell line HeLa (ATCC catalog # CCL-2), the human liver cell line HepG2 (ATCC catalog # HB-8065), the human laryngeal cancer cell line HEp-2 (ATCC catalog # CCL-23), the human rhabdomyosarcoma cell line RD (ATCC catalog # CCL-136) and the human kidney cell line HEK-293 (ATCC catalog # CRL-1573) were maintained in DMEM or RPMI 1640 (Life Technologies, CA, USA) supplemented with 10% fetal bovine serum (Life Technologies) plus 100 IU of penicillin and 100 µg of streptomycin per ml at 37°C in the presence of 5% CO_2_.

### Viruses

HAdV-B55 strain Y16/SX/2011 was isolated in Taiyuan city, Shanxi province, China, in 2012. Swab samples from a patient with febrile respiratory infectious illness were collected and subjected to viral isolation in A549 cells. The presence of HAdV-B55 strain Y16/SX/2011 was confirmed by whole-genome sequencing (GenBank accession number KF911353).

HAdV-B11 prototype strain Slobitski (GenBank accession number NC_011202, ATCC catalog # VR-12) and HAdV-B14 prototype strain de Wit (GenBank accession number AY803294, ATCC catalog # VR-15) were obtained from the ATCC. All viral stocks were prepared in A549 cells, and the viral titers were measured using a standard plaque assay.

### Plaque assay

A standard plaque assay was used to determine the viral titers. The assay was performed using 100% confluent A549 cells in 12-well plates. Briefly, A549 cells were inoculated with viruses and incubated for 2 h at 37°C. The monolayers were then washed twice with PBS, overlaid with 1 ml of 2% low-melting-point agarose (Promega, WI, USA) mixed 1∶1 with 2×1640 (20% FBS plus 200 IU of penicillin and 200 µg of streptomycin per ml), and placed in a 37°C incubator with 5% CO_2_ for 6 d. Six days post-infection, the plates were fixed with 4% formaldehyde and stained with a 1% (wt/vol) crystal violet solution. The plaques were counted, and the titers were calculated and expressed in PFU/ml.

### Cell infectivity

The infectivity of these three human adenoviruses in various cells of distinct origins was evaluated. Six human cell lines, including A549, HeLa, HepG2, RD, HEp-2 and HEK-293, were separately infected with HAdV-B55, HAdV-B11 or HAdV-B14 at a multiplicity of infection (MOI) of 0.1 in RPMI 1640 or DMEM with 2% FBS and incubated at 37°C for 2 h, after which the inoculum was removed and replaced with fresh RPMI 1640 or DMEM with 2% FBS. The development of cytopathic effects (CPEs) was observed daily. The titers of viruses released into the cell supernatant were determined by plaque assays, whereas the viral loads in the cells were determined by real-time quantitative PCR (qPCR) methods.

### Growth kinetics assay

HEp-2 cells in DMEM containing 2% FBS were infected with viruses at an MOI of 0.1 and incubated at 30°C. A549 cells in RPMI 1640 containing 2% FBS were infected with viruses at an MOI of 0.1 and incubated at 37°C. Supernatants and cells were harvested at 1, 3, 5 and 7 d post-infection. Viral titers were determined by plaque assays using the A549 cells or by real-time qPCR assays.

### Cell viability assay

Cell viability was determined using an MTS Cell Proliferation Assay (Promega) according to the manufacturer's protocol. Briefly, 5×10^3^ cells were seeded in each well in a standard 96-well plate and incubated at 37°C with 5% CO_2_. After overnight culture, the RPMI 1640 media was removed, and the cells were infected with virus. Infected and uninfected cells were assayed at 12, 24 and 48 h post-infection. The absorbance was determined at 490 nm using a Beckman microplate spectrophotometer.

### Apoptosis assay

A549 cells in 96-well plates were infected with viruses at an MOI of 10 and then incubated at 37°C. Caspase-3/7 activities at 6 and 12 h post-infection were tested with an ApoTox-Glo Triplex Assay (Promega). Luminescence was detected with the plate reader SpectraMax M5 (Molecular Devices, CA, USA). The apoptosis assay was repeated four times, and uninfected cells were used as the negative control.

### Thermostability analysis

All viruses were incubated at 37°C for various numbers of weeks. After incubation at 37°C, the viruses were quickly cooled in ice water and stored at −80°C until titer determination. The loss of viral infectivity was determined using a standard plaque assay on A549 cells. Thermostability was calculated as the slope of the regression line. A large slope indicated that the adenovirus was less stable at the experimental temperature.

### Viral DNA extraction and real-time qPCR

Cells were homogenized, and then viral DNA was extracted using a QIAamp DNA Mini Kit (Qiagen, Hilden, Germany). The viral loads were quantified by using HAdV type-specific real-time qPCR assays and a SYBR Premix Ex Taq Kit (Takara, Dalian, China) according to the manufacturer's instructions. The following cycling conditions were used in the LightCycler 2.0 system (Roche, Penzberg, Germany): an initial denaturing cycle at 95°C for 20 s, followed by 40 cycles of amplification (95°C for 5 s and 60°C for 20 s; the fluorescence was recorded at 60°C) and then a melting curve analysis cycle (95°C for 0 s, 65°C for 15 s and 95°C for 15 s, with the temperature slowly increased by 0.1°C/s). The primers used in this study are listed in Table 1. The 10-fold serial dilutions of viruses that had been quantitated by the plaque-forming assay were used as standard samples for standard curve analysis (Figure S1).

**Table 1:** Primers used in the real-time qPCR assay.

Type	Primer	Sequence (5′-3′)
HAdV-B55	ADV55-SYBR-F	AGATGAAGAAAGTAAACCGATTT
	ADV55-SYBR-R	CCATCAAGGTCAGTCCAA
HAdV-B11	ADV11-SYBR-F	AGCAGCAGCAGCAATCACA
	ADV11-SYBR-R	GGCAGTTGCAGTAGTTGC
HAdV-B14	ADV14-SYBR-F	TTGGATAAAGGGGTTGAAACTAC
	ADV14-SYBR-R	TCCCCGTCTTCATTTTGC

### Statistical analysis

Statistical tests were performed with GraphPad software, using Student's *t*-test, two-way ANOVA and the Chi-square test, as indicated in the figure legends.

## Results

### Distinct cell susceptibility to HAdV-B55 and its parental viruses

HAdV-B55 was derived from homologous recombination between HAdV-B11 and HAdV-B14 [2], and the potential biological relationships between these three types of adenoviruses are therefore of great interest. Here, a series of human cell lines that are usually used for HAdV infections were initially infected with HAdV-B55 and its parental viruses, HAdV-B11 and HAdV-B14. As shown in Table 2, all cells, including the A549, HeLa, HepG2, RD, HEp-2 and HEK-293 cell lines, were susceptible to infection with HAdV-B55, HAdV-B11 and HAdV-B14. Complete CPEs were observed at 5 d post-infection in all cell types tested. However, the CPE induced by each HAdV varied. HAdV-B11 led to the rapid development of a complete CPE in all cell lines except the RD cells within 2 d post-infection. The CPE development of HAdV-B14 was similar to that of HAdV-B11; however, no typical CPE was observed in the HeLa cells. In contrast to HAdV-B14 and HAdV-B11, the CPE development caused by HAdV-B55 in all cell lines was much slower. HAdV-B55 failed to cause a CPE in any cell type within 2 d post-infection, except mild CPE development in the 293 cells.

**Table 2:** Differential cell-line susceptibility to HAdV-B55, HAdV-B14 or HAdV-B11 in terms of the CPE.

Cell line	CPE^a^: 2 dpi			5 dpi		
	HAdV-B11	HAdV-B14	HAdV-B55	HAdV-B11	HAdV-B14	HAdV-B55
A549	4	4	N	4	4	4
RD	N	N	N	4	4	4
HeLa	4	N	N	4	4	4
HEp-2	4	4	N	4	4	4
HepG2	4	4	N	4	4	4
HEK-293	4	4	1	4	4	4

a Grading of the extent of the CPE: N  =  negative, 1 = 1–25%, 2 = 26–50%, 3 = 51–75% and 4 = >75%. The data are representative of three independent experiments.

**Figure 1:**
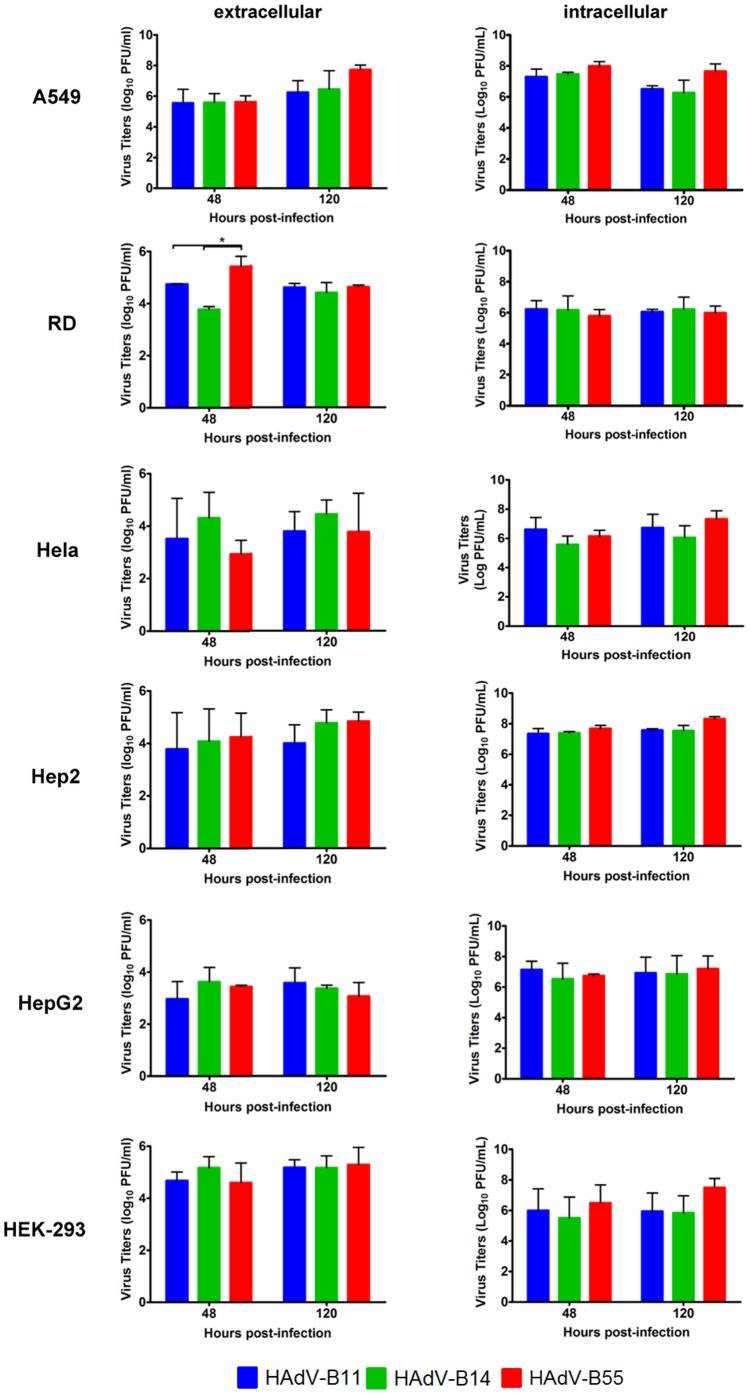
Differential cell line susceptibility to HAdV-B55, HAdV-B14 and HAdV-B11. Viral titers (log_10_ PFU/ml) of HAdV-B11 (blue), HAdV-B14 (green) and HAdV-B55 (red) in RD, HeLa, HEp-2, HepG2 and HEK-293 cells at 2 and 5 d post-infection. Three independent experiments were performed. The data are shown as the mean ± standard deviation. The statistical significance of the results was determined using two-way ANOVA tests, *P<0.05.

In the following experiments, the replication efficiencies of the three adenoviruses were characterized in the six cell lines mentioned above (Figure 1). All adenoviruses had detectable viral loads in the supernatants. Interestingly, although no profound CPE was observed in the cells within 48 h post-infection, HAdV-B55 replicated to a titer similar to that of HAdV-B11 or HAdV-B14. The titers of the three adenoviruses released into the supernatant at 48 h post-infection were approximately 10^4^ PFU/ml in the HeLa, HEp-2 and HepG2 cells, whereas peak titers of approximately 10^5^ PFU/ml were determined in the A549 and HEK-293 cells. Although HAdV-B55 exhibited higher replication in the RD cells compared with that of HAdV-B11 at 48 h post-infection, there was no significant difference observed in any type of cell between HAdV-B11, HAdV-B14 and HAdV-B55 at 120 h post-infection. Moreover, the intracellular viral titers were determined by qPCR at 48 and 120 h post-infection. The three adenoviruses exhibited similar replication efficiencies in all cell types. Moreover, as shown in Figure 1, HAdV-B55, HAdV-B11 and HAdV-B14 all replicated at a higher rate, with titers of approximately 10^8^ PFU/ml, in the A549 and HEp-2 cells. These results suggested that HAdV-B55 and its parental viruses have different cell susceptibilities and replication features.

### Viral fitness in A549 and HEp-2 cells

**Figure 2:**
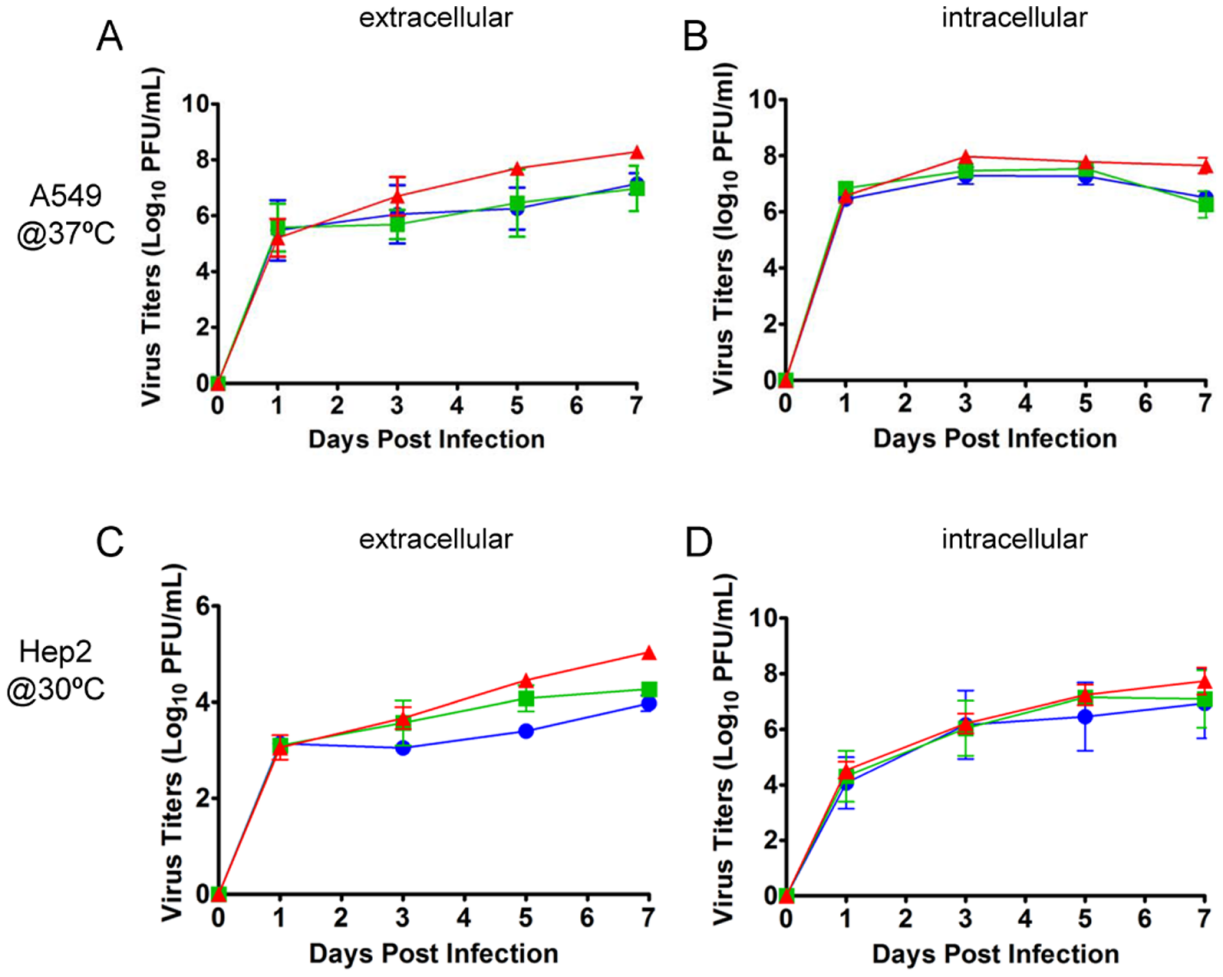
Viral growth kinetics of HAdV-B55, HAdV-B14 and HAdV-B11 in A549 and HEp-2 cells. A549 (A & B) and HEp-2 (C & D) cells were infected with HAdV-B11 (blue), HAdV-B14 (green) or HAdV-B55 (red) at an MOI of 0.1, and the supernatants and cells were separately harvested at 1, 3, 5 and 7 d post-infection. The supernatants samples were titrated by a plaque assay on A549 cells. Viral titers in the cell samples were determined by qPCR. The data are shown as the mean ± standard deviation of three independent experiments. A & C, the extracellular viral loads in the supernatants. B & D, the intracellular viral loads in the cells.

Here, the viral fitness of HAdV-B55, HAdV-B11 and HAdV-B14 in the lower-airway cell line A549 and the upper-airway cell line HEp-2 was analyzed, and growth kinetics assays were performed at different temperatures. The HEp-2 cells were infected at 30°C to simulate the environment of the upper respiratory system, whereas the A549 cells were infected at 37°C to simulate the environment of the lower respiratory system. As shown in Figure 2, there were significant differences between the three adenoviruses in terms of their growth kinetics. HAdV-B55 replicated to a peak extracellular titer of approximately 10^8^ PFU/ml in the A549 cells and approximately 10^4^ PFU/ml in the HEp-2 cells. These values were significantly higher than those of HAdV-B14 and HAdV-B11. The viral titers of the three adenoviruses in the HEp-2 cells at a low temperature (30°C) were remarkably lower than those in the A549 cells at a slightly higher temperature (37°C) (Figure 2C). Interestingly, there was no observable difference in the growth kinetics of HAdV-B11 and HAdV-B14 in the A549 cells at 37°C; however, HAdV-B14 exhibited a higher growth rate than did HAdV-B11 in the HEp-2 cells at 30°C. Similar to the data on the growth characteristics, the intracellular growth kinetics of the three adenoviruses showed that HAdV-B55 had slightly higher viral loads at 120 h post-infection in the A549 cells (Figures 2B & 2D). These results indicated that HAdV-B55 has greater viral fitness in both upper and lower respiratory tract cells than do either HAdV-B11 or HAdV-B14.

### Cytotoxicity and apoptosis analyses in A549 cells

**Figure 3:**
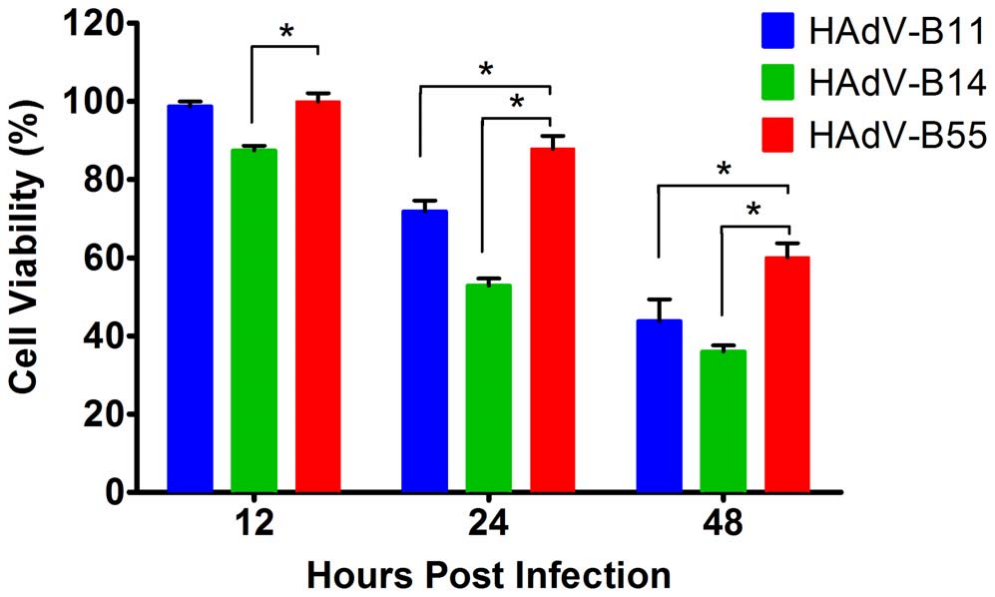
Toxicity of HAdV-B55, HAdV-B14 and HAdV-B11 in A549 cells. A549 cells were infected with one of the three viruses at an MOI of 10. At 12, 24 and 48-infection, the results were assessed using MTS Cell Proliferation reagent (Promega) according to the manufacturer's instructions. The resulting values (the ratio of infected to uninfected cells) are presented as the mean ± standard deviation of four replicates. These results are representative of two independent experiments. The asterisks indicate statistically significant (P<0.001) differences, as determined using two-way ANOVA tests.

To further understand the unique biological features related to pathogenesis, the differential cytotoxicity levels induced by the three human adenoviruses were compared in the A549 cells. As shown in Figure 3, HAdV-B55 was less cytotoxic in the A549 cells than was HAdV-B11 or HAdV-B14. Only in the early stage of infection (at 12 h post-infection) did HAdV-B11 exhibit similar toxicity (98.7%) to HAdV-B55 (99.8%), whereas HAdV-B14 showed slightly higher toxicity (87.4%). HAdV-B55 exhibited slight toxicity (60.1%) even in the later stages of infection, in contrast to HAdV-B11 and HAdV-14, which exhibited more significant toxicity (43.8% and 36.0%, respectively).

**Figure 4:**
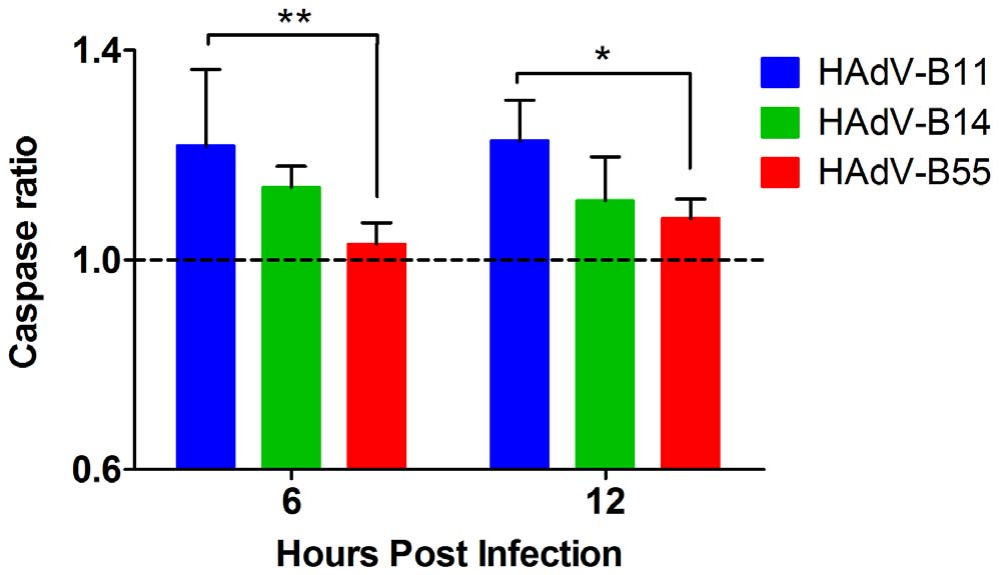
Levels of apoptosis in HAdV-B55-, HAdV-B14- or HAdV-B11-infected A549 cells. A549 cells were infected with HAdV-B11, HAdV-B14 or HAdV-B55 at an MOI of 10. At 12, 24 and 48 h post-infection, the results were assessed using the ApoTox-Glo Triplex Assay (Promega) according to the manufacturer's instructions. The values of the caspase ratio (infected to uninfected) are shown as the mean ± standard deviation of four replicates from two independent experiments. Statistical significance was determined using a two-way ANOVA test. **P<0.01; *P<0.05.

Furthermore, virus-induced apoptosis was analyzed by measuring caspase-3/7 activities during the early stages of infection. Our results showed that the ratio of caspase-3 to caspase-7 in the HAdV-B11-infected A549 cells was significantly higher than in the HAdV-B55-infected cells at both time points tested (Figure 4). The ratio of caspase-3 to caspase-7 in the HAdV-B55-infected cells was 1.03 at 6 h post-infection and 1.08 at 12 h post-infection, indicating that HAdV-B55 induced a low level of apoptosis in the A549 cells. These results suggested that compared with HAdV-B11 and HAdV-B14, which induced severe apoptosis in the A549 cells, HAdV-B55 infection resulted in mild apoptosis during the early stages of infection. The observed low virus-induced cytotoxicity and lack of CPE development despite viral infection suggest that certain viral factors of HAdV-B55 are involved in reducing its induced cytopathology and cell death.

### Thermal sensitivity analysis

**Figure 5:**
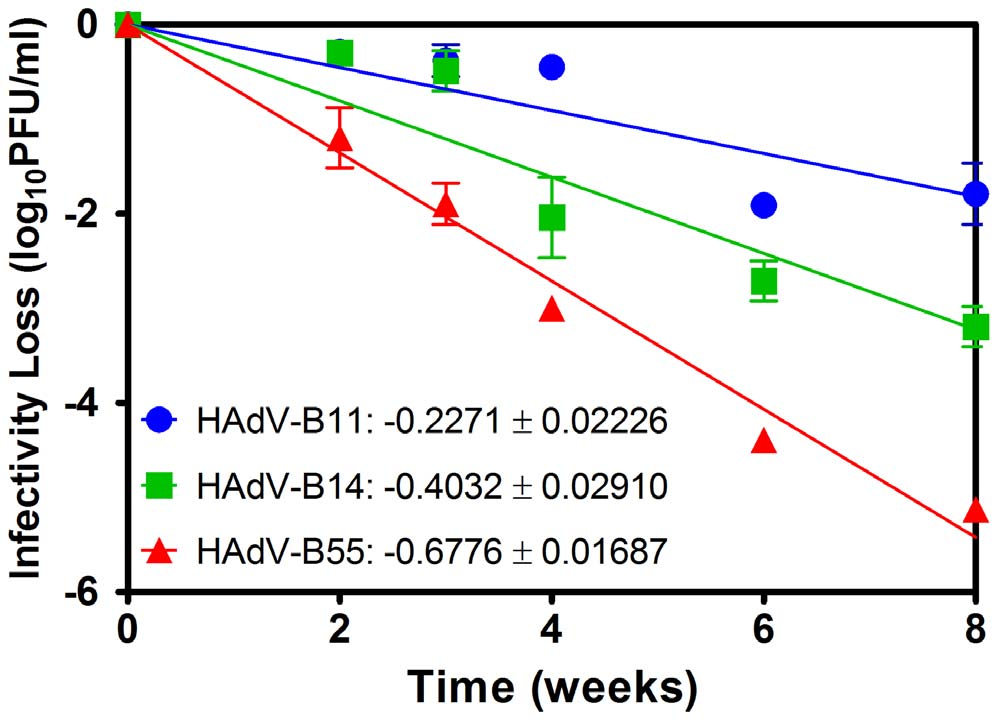
The effect of heat treatment on the infectivity of HAdV-B55, HAdV-B14 and HAdV-B11. The viruses were incubated for 2, 3, 4, 6 or 8 weeks at 37°C. Viral titers in heat-treated virus samples were determined by plaque assays on A549 cells. Each value represents the mean ± standard deviation of two separate experiments.

Temperature is known to play an important role in respiratory viral transmission [18], [19]. For this reason, the thermal stabilities of HAdV-B55, HAdV-B11 and HAdV-B14 were analyzed by determining their rates of infectivity at 37°C. HAdV-B55 had lost most of its infectivity after 8 weeks, with a 4.8 log10 decrease in its titer, whereas HAdV-B14 exhibited significant tolerance to heat treatment, with a 3.0 log10 decrease in its titer. HAdV-B11 was the most resistant virus, with only a 1.5 log10 decrease under the same conditions. As shown in Figure 5, the slope of the regression line for HAdV-B55 (−0.6776±0.01687 log10 PFU/week) indicated that it had lower stability than did HAdV-B11 (−0.2271±0.02226 log10 PFU/week) and HAdV-B14 (−0.4032±0.02910 log10 PFU/week). These results showed that HAdV-B55 is less thermostable than either HAdV-B11 or HAdV-B14 is under heat treatment, which may limit the ability of HAdV-B55 to be transmitted between hosts.

## Discussion

HAdV-B55 has been identified to be a recombinant adenovirus derived from HAdV-B14 and HAdV-B11 [2], [3]. The potential effect of homologous recombination on the specific tropism and pathogenicity of HAdV-B55 is not well understood and is of public health concern. In the current research, a comparison study assessing cell tropism and infectivity, viral replication, cytotoxicity, apoptosis and thermostability was performed to compare HAdV-B55 with HAdV-B11 and HAdV-B14. To compare the three viruses, HAdV-B55, HAdV-B11 and HAdV-B14 were passaged only once in A549 cells to prepare stocks. The results of the cell tropism assay on six human cell lines from different tissues and organs showed that HAdV-B55 exhibited similar cell tropism to HAdV-B11 and HAdV-B14, although the first and third infect the respiratory tract and the second infects the urinary tract, causing substantially different clinical symptoms. The data on cellular infectivity were consistent with the results of previous studies, indicating that the epithelial cell lines HEK-293 and A549 are optimal cell lines for in vitro HAdV-B14 propagation [20], [21]. Furthermore, the high viral loads recorded in the airway cell lines for all three adenoviruses revealed the viruses' potential risk of causing respiratory infectious diseases in which co-infection could lead to subsequent homologous recombination. HAdV-B55 did not produce detectable CPE development by 2 d post-infection and exhibited less toxicity. It was helpful to observe the viral replication in the early stages of infection. In the A549 cells, the similar intracellular replication of the three adenoviruses suggested that these viruses had similar replication efficiencies inside the cells. In contrast, the higher viral load of HAdV-B55 than of HAdV-B11 or HAdV-B14 3 d post-infection may have resulted from differences in the release efficiency of the virus particles. The high viral replication levels of HAdV-B55 in lower respiratory cells may be a risk factor, resulting in higher rates of viral pneumonia and hospitalization. However, the low thermostability of HAdV-B55 may limit its human-to-human transmission. Although the cultured cell lines used in the study may not resemble the original tissues and organs from which they were derived, these results still provide useful fundamental information that is important for future viral isolation and identification in virology and clinical laboratories.

Previous studies revealed that HAdV-B11 and HAdV-B14 have similar receptors [22]–[24]. In contrast to other species of HAdV, most species of B adenoviruses use CD46 and/or desmoglein-2 (DSG-2) as their primary attachment receptor [23], [24]. The current study also provides information on the distinct infectivity of the three viruses, which may reveal differences in the cellular receptor binding of HAdV-B11 and HAdV-B14. A previous study showed that HAdV-B11 and HAdV-B14 both use CD46 as their cellular attachment receptor; however, the levels of attachment of HAdV-B11 to CD46 were significantly higher than those of HAdV-B14 [24]. In a recent study, DSG-2 was identified as the primary high-affinity receptor used by adenoviruses Ad3, Ad7, Ad11 and Ad14, although DSG-2 does not completely block HAdV-B11 from approaching cells [23]. These differences highlight the complexity of the interaction between the virus particles and their cellular receptors. HAdV-B55 has the same fiber protein as HAdV-B14 does, although the cellular binding activity of HAdV-B55 was found to be significantly lower than that of HAdV-B14 (data not shown). A previous study also revealed that the fiber protein does not completely block adenovirus attachment to target host cells [22]. These findings suggest that hexon or penton genes other than that of the fiber may play a role in the interactions between the viral and the cellular receptors. Recombination in the hexon gene may also affect the attachment of HAdV-B55 to host cells. The current study also revealed that HAdV-B55 exhibited cell tropism similar to HAdV-B14 but not HAdV-B11.

Although it remains unclear whether HAdV-B is primarily transmitted through the droplet, airborne or contact mode of transmission, the thermostability of human adenovirus in the environment contributes to its transmission. The effect of temperatures on viral replication was observed in the current study. We used two cell lines cultured at different temperatures to simulate the different physiological environments of the upper and lower respiratory tracts. Our results showed that HAdV-B55 and HAdV-B14 both exhibited higher replication efficiencies than did HAdV-B11, which suggests that HAdV-B14 and HAdV-B55 may be transmitted by respiratory droplets. Furthermore, the heat stability of HAdV-B55 is lower than that of HAdV-B14, which may explain why fewer HAdV-B55 infections have been reported in the population. The thermostability of a virus commonly depends on the structure of the viral proteins. Penton and hexon proteins are two very important structural proteins for the formation of the virus particle. Homologous recombination in the hexon gene may change and even destroy the conformation of the hexon protein and virus particle, thus affecting viral stability. However, our results also reflected the critical role of the penton protein in maintaining the thermostability of an adenovirus.

Based on this comparison of the biological characteristics of these three distinct adenoviruses, we have learned about the differences between HAdV-B55 and HAdV-B11/14 in terms of their viral replication efficacy, cytotoxicity and apoptosis induction. These data suggest that HAdV-B55 possesses distinct biological characteristics, although it has a similar cell tropism as its parental adenoviruses. This study provides important foundational information about HAdV-B55, which is helpful for understanding this re-emergent acute respiratory pathogen. Furthermore, these findings may be helpful for understanding the molecular pathogenesis of HAdV-B55 and may contribute to the development of novel antiviral treatments for HAdV-B55 infection.

## Supporting Information

Figure S1 **The standard curves of qPCR for HAdV-B11 (A), HAdV-B14 (B) and HAdV-B55 (C), respectively.** Real-time PCR assays were performed using viral DNAs for each assay. The 10-fold serial dilutions of viruses were used as standard samples for standard curve analysis. The slope, the Y-intercept and the R2 value were determined.
(DOCX)
