# Supplementary material for: In Vitro Characterization of Human Adenovirus Type 55 in Comparison with Its Parental Adenoviruses, Types 11 and 14
Source: PLoS One. 2014 Jun 23;9(6):e100665. doi: 10.1371/journal.pone.0100665 (PMC4067339; doi:10.1371/journal.pone.0100665)
Supplement: Figure S1 — The standard curves of qPCR for HAdV-B11 (A), HAdV-B14 (B) and HAdV-B55 (C), respectively. Real-time PCR assays were performed using viral DNAs for each assay. The 10-fold serial dilutions of viruses were used as standard samples for standard curve analysis. The slope, the Y-intercept and the R2 value were determined. (DOCX) [file pone.0100665.s001.docx]

**Supporting Information**

A

B

C

Figure S1.

The standard curves of qPCR for HAdV-B11 (A), HAdV-B14 (B) and HAdV-B55 (C), respectively.

Real-time PCR assays were performed using viral DNAs for each assay. The 10-fold serial dilutions of viruses were used as standard samples for standard curve analysis. The slope, the Y-intercept and the R^2^ value were determined.
